# Adsorption mechanism of Cr(VI) onto GO/PAMAMs composites

**DOI:** 10.1038/s41598-019-40344-9

**Published:** 2019-03-06

**Authors:** Han Liu, Fan Zhang, Zhiyuan Peng

**Affiliations:** 2011 Cooperative Innovation Center of industrial technology for manganese, zinc and vanadium of Hunan Province, Hunan Provincial Engineering Laboratory of Integrated Control and Remediation of Heavy Metal Pollution from Mn-Zn Mining, National Demonstration Center for Experimental Chemistry Education, Jishou University, Jishou, Hunan 416000 China

## Abstract

Graphene oxide/polyamidoamine dendrimers (GO/PAMAMs) composites were used to remove Cr(VI) from simulated effluents, the adsorption kinetics and thermodynamics of Cr(VI) onto GO/PAMAMs were systematically investigated. The results showed that the optimum pH value was 2.5, the removal percentage reached 90.7% for 30 mg/L of Cr(VI) within 120 min. The adsorption process was well described by pseudo-second-order kinetic model. The maximum adsorption capacities of Cr(VI) onto GO/PAMAMs were found to be 131.58, 183.82 and 211.42 mg/g at 293.15, 303.15 and 313.15 K, respectively, which were calculated from the Langmuir model equation. The adsorption thermodynamic parameters indicate that the adsorption of Cr(VI) onto GO/PAMAMs is a spontaneous endothermic process. The XPS analysis reveals the adsorption and removal mechanism of Cr(VI) on GO/PAMAMs that first the Cr(VI) binds to the protonated amine of GO/PAMAMs, then Cr(VI) be reduced to Cr(III) with the assistance of π-electrons on the carbocyclic six-membered ring of GO in GO/PAMAMs, and then Cr(III) was released into solution under the electrostatic repulsion between the Cr(III) and the protonated amine groups.

## Introduction

Chromium is widely used in the industries of leather-tanning^1^, mining, textile dyeing, manufacturing processes of anti-corrosion agents and pigments^2^, which means that it’s unavoidable to generate large amounts of chromium-containing wastewater. As is known that hexavalent [Cr(VI)] and trivalent [Cr(III)] are the most stable forms in aquatic environment and Cr(VI) is about 1000 times more poisonous than Cr(III)^3^. Due to its highly poisonous and extremely mobile in the surface-water and groundwater in a broad pH range, Cr(VI) has been identified as a potential carcinogenic substance by the Environmental Protection Agency of USA. Long-term exposure to Cr(VI) can lead to nose perforation, skin ulceration, liver damage and lung cancer^4,5^. Hence, Cr(VI) has been placed as one of eight prior toxic chemical substances listed by the World Health Organization (WHO), and the maximum amount of Cr(VI) for discharging to inland surface water must be restricted to 0.2 mg/L according to the industrial wastewater discharge standard, which set by the Ministry Environmental protection of China^6^, meanwhile the guideline of Cr(VI) content for drinking water established by the WHO must be below 0.05 mg/L^4^.

Various methods have been investigated to remove Cr(VI) from industrial wastewater, such as electro-coagulation^7^, photo-catalytic reduction^8,9^, membrane separation^10,11^, adsorption^12–14^, ion-exchange^15,16^ and microbial remediation^17,18^. Among these methods, adsorption has won a great deal of concern due to it’s a series of advantages, such as cost-effective, high sorption capacity, simple operation and no secondary pollution. A variety of adsorbents, such as activated carbons^19^, nanotubes^20^, clays^21^, functionalized tannin^22^, carbonate plant^23^ and iron nanoparticle composites^24,25^ had been studied for removing Cr(VI) from wastewater. Graphene oxide (GO) has large specific area and multi-functions groups, such as carboxyl, hydroxyl and epoxy on its surface, which is benefited to binding with heavy metals. GO and its composites have been investigated to remove some heavy metal ions, such as Pb(II), Cd(II), Cu(II) and Hg(II)^26,27^. However, removing Cr(VI) only by the graphene oxide is inefficient due to the limitation of functional groups and easy to aggregate, which restrict its large scale application on wastewater purification. So some chemical scholars tried to prepare modified GO material for adsorption of heavy metal ions. For example, Hui-Ling Ma *et al*.^28^. had prepared ethylenediamine reduced GO (ED-RGO) for chemical reduction and removal of Cr(VI) from acid aqueous solution and he had found that ED-RGO could effectively reduce Cr(VI) to low toxic Cr(III) at low pH. Xiaoshu Lv *et al*.^29^. synthesized nanoscal zero-valent iron (nZVI) assembled on magnetic Fe_3_O_4_/graphene (nZVI@MG) nanocomposites to remove Cr(VI) from aqueous solution, experimental results showed that the removal efficiency of Cr(VI) reached 83.8%, which was much higher than those of the individual (nZVI, Fe_3_O_4_ NPs and graphene). Hou Wang *et al*.^30^. fabricated a novel ternary magnetic composites consisted of reduced graphene oxide (rGO), polypyrrole (Ppy) and Fe_3_O_4_ nanoparticles (Ppy-Fe_3_O_4_), the Ppy-Fe_3_O_4_/rGO nanohybrid exhibited excellent adsorption performance for Cr(VI), the maximum adsorption capacity for Cr(VI) onto Ppy-Fe_3_O_4_/rGO reached 293.3 mg/g, which was much higher than that of Fe_3_O_4_/rGO, the adsorption mechanism was through both electrostatic attraction and ion exchange, meanwhile XPS analysis revealed that Cr(VI) was reduced to low poisonous Cr(III) by nitrogen species of Ppy.

In this paper, we used 2.0 G GO/PAMAMs composites, which were prepared by grafting 2.0 G PAMAM to GO in our previous study^31^. The influence factors such as pH value, initial Cr(VI) concentration, temperature and contact time were investigated. During the investigation process, we focused on studying the kinetics and thermodynamics of Cr(VI) adsorption onto GO/PAMAMs. The adsorption kinetics was investigated by pseudo-first, pseudo-second equation and intraparticle diffusion model. In order to obtain the *q*_*m*_ values of Cr(VI) adsorption on PAMAMs-GO, the Langmuir and Freunlich model are used for fitting the adsorption isotherm data. To investigate the influence of temperature on the thermodynamic parameters, thermodynamic parameters, e.g. Δ*H*^*0*^, Δ*S*^0^and Δ*G*^0^ for the adsorption of Cr(VI) on PAMAMs-GO were also calculated. The effect of Cl^−^ and SO_4_^2−^ on the adsorption of Cr(VI) by GO/PAMAMs and XPS analysis were especially explored in the paper.

## Results and Discussion

### Characterization

The GO/PAMAMs were analyzed by FT-IR, TGA Raman spectra, the Surface Area Analyzer in our published paper^31^. The BET specific surface area of GO/PAMAMs is 25 m^2^/g, which was much lower than that of previous graphene oxide sample data^31^. This might be due to the incomplete exfoliation of graphene oxide and the agglomerations occurred during preparation process^31^.

**Figure 1:**
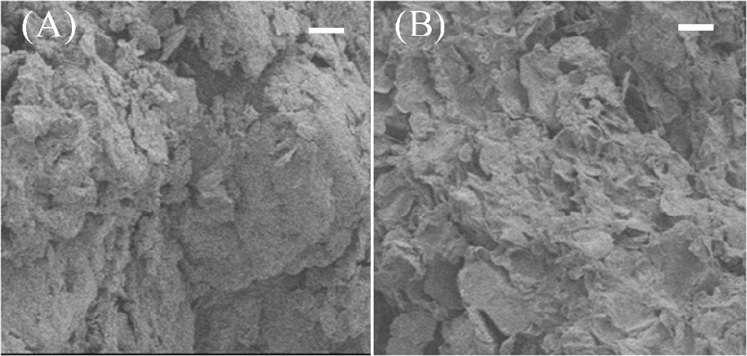
SEM images of GO/PAMAMs (**A**) and GO/PAMAMs adsorbed Cr(VI)) (**B**). (the bar is 5 um).

Figure 1 showed that the surface morphology of GO/PAMAMs was changed after adsorbing Cr(VI). It was easily found that the GO/PAMAMs had relatively fluffy and foam-like structures, which certainly provide good platform for heavy metal adsorption. After adsorbing Cr(VI), the GO/PAMAMs became flakes but retained its fluffy characteristic, this may favor the regeneration of GO/PAMAMs as an adsorbent for Cr(VI) adsorption.

### Effect of pH on removal of Cr(VI)

**Figure 2:**
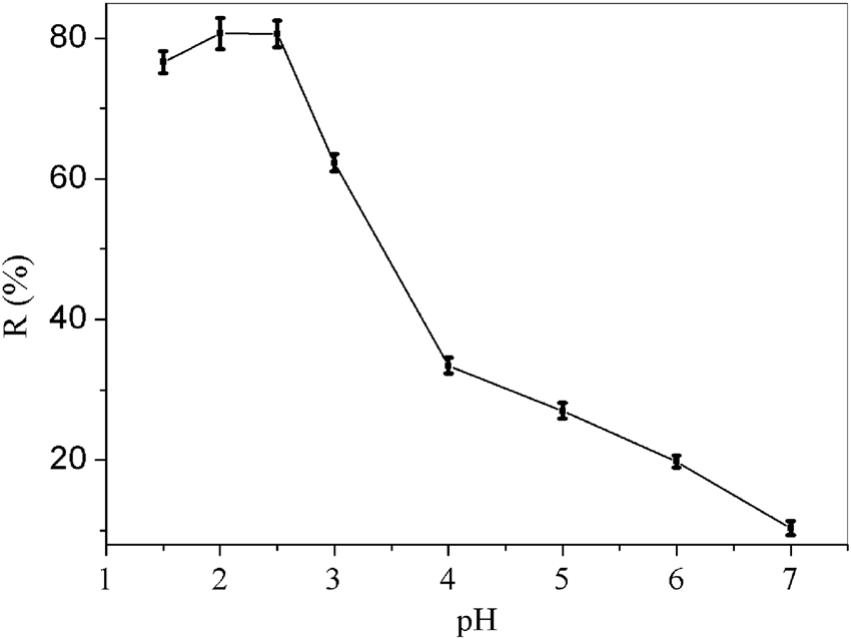
The curve of Cr(VI) removal efficiency versus pH.

As the pH value has a significant effect on most adsorbents for removal of heavy metals, so it should be given priority consideration. As depicted in Fig. 2, there is a relatively high removal efficiency for Cr(VI) in the pH range of 1.5–2.5, the maximum removal efficiency for Cr(VI) reached 80.70% at pH = 2.5. While in the pH range of 2.5–7.0, there is a sharp decline in removal efficiency of Cr(VI), the removal efficiency of Cr(VI) decrease as the increase in pH. The highly dependence on pH of Cr(VI) adsorption onto the GO/PAMAMs can be explained as follows: the most probable species of Cr(VI) present in aqueous solution are Cr_2_O_7_^2−^, CrO_4_^2−^, HCrO_4_^−^ and H_2_CrO_4_, which depend on the solution pH, Cr(VI) concentration and redox potential^32^. In the acid medium with pH ranging from 1.0 to 4.0, HCrO_4_^−^ is the major species of Cr(VI)^32^, and the surface of GO/PAMAMs was surrounded by adequate H^+^. Hence, the amine groups of the adsorbent were easily protonated and positively charged, which promoted the approach of negatively charged Cr(VI) species (HCrO_4_^−^) attributed to the electrostatic interaction as Eq. (1). This point was supported by the curve of zeta potentials of GO/PAMAMs versus pH values in our published literature^31^. While in the relatively neutral solution with pH ranging from 4.0 to 7.0, CrO_4_^2−^ is the predominant species, which means that adsorbing one Cr(VI) anion must consume two active sites (showed as Eq. (2)) on GO/PAMAMs compared with that of HCrO_4_^−^ species^6^. Meanwhile, due to the lacking of proton, there are few protons to form protonated amine groups for adsorption of CrO_4_^2−^ species. Therefore, low pH medium is favorable for the removal of Cr(VI). Since at pH = 2.5, the GO/PAMAMs has the maximum removal efficiency, so we choose pH = 2.5 as the optimal pH medium for adsorption of Cr(VI) in the following experiments

1 $${\rm{R}}\,{({{\rm{NH}}}_{2})}_{{\rm{n}}}\mathop{\longrightarrow }\limits^{{{\rm{H}}}^{+}}{\rm{R}}\,{{(\mathrm{NH}}_{{\rm{3}}}^{+})}_{{\rm{n}}}\mathop{\longrightarrow }\limits^{{{\rm{HCrO}}}_{4}^{-}}{\rm{R}}\,{{[\mathrm{NH}}_{{\rm{3}}}^{+}{{\rm{HCrO}}}_{4}^{-}]}_{{\rm{n}}}$$

2 $${\rm{2R}}\,{({{\rm{NH}}}_{2})}_{{\rm{n}}}\mathop{\longrightarrow }\limits^{{{\rm{H}}}^{+}}{\rm{2R}}\,{{(\mathrm{NH}}_{{\rm{3}}}^{+})}_{{\rm{n}}}\mathop{\longrightarrow }\limits^{{{\rm{CrO}}}_{4}^{2\,-}}[\mathrm{2R}(\,-\,{{\rm{NH}}}_{{\rm{3}}}^{+}{)}_{{\rm{n}}}{{\rm{nCrO}}}_{4}^{2\,-}]$$

where R represents the skeleton of GO/PAMAMs, the value of n is between 1 to 8.

### Effect of contact time and adsorption kinetics

**Figure 3:**
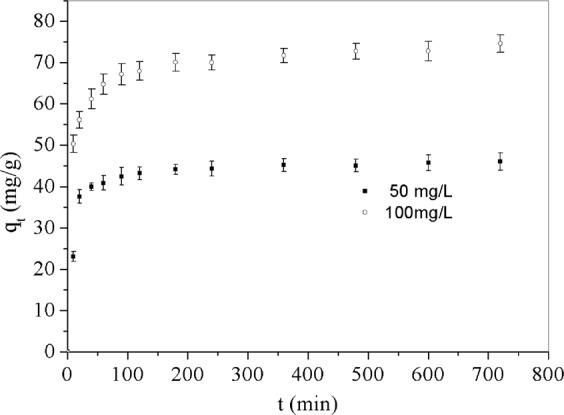
The equilibrium Cr(VI) adsorption curve of *q*_*t*_ versus *t*.

In order to obtain the minimum contact time for equilibrium adsorption, the time-dependent behavior of Cr(VI) adsorption of two different initial concentration (50 and 100 mg/L) were carried out in the time range of 10–720 min as shown in Fig. 3. From the Fig. 3 we can be observed that the adsorption rate was very quick for the first 20 minutes and then became slow until nearly zero. For both *C*_0_ = 50 and 100 mg/L of Cr(VI), 180 min of contact time is enough to reach equilibrium of adsorption at 303.15 K. Generally, the pseudo-first-order equation (3) and pseudo-second-order equation (4)^4^ are utilized to investigate the adsorption kinetics, and usually the Eq. (4) offered a better description of adsorption kinetics. Besides, the Intraparticle model is also the most widely used model to describe the kinetic mechanism of the adsorption process since it gives information of the diffusion of Cr(VI) anions onto GO/PAMAMs particles in the liquid adsorption system, the most commonly used intraparticle diffusion model is expressed in Eq. (5)^33^. In this paper, pseudo-first, pseudo-second equation and intraparticle diffusion model were used to fit the adsorption data.

3 $${\rm{The}}\,{\rm{pseudo}} \mbox{-} {\rm{first}} \mbox{-} {\rm{order}}:\,\mathrm{ln}(\frac{{q}_{e}-{q}_{t}}{{q}_{e}})=-\,{k}_{1}t$$

4 $${\rm{The}}\,{\rm{pseudo}}-{\rm{second}}-\,{\rm{order}}:\frac{t}{{q}_{t}}=\frac{1}{{q}_{e}}t+\frac{1}{{k}_{2}{q}_{e}^{2}}$$

5 $${\rm{The}}\,{\rm{Intraparticle}}\,{\rm{diffusion}}\,{\rm{model}}:{q}_{t}={k}_{i}{t}^{1/2}+C$$

where *q*_*e*_ and *q*_*t*_ are the mass of Cr(VI) adsorbed (mg/g) at equilibrium and time *t*, respectively, *k*_1_ is the first-order reaction rate constant of adsorption (min^−1^), and *k*_2_ is the pseudo-second order reaction rate constant (g.mg^−1^.min^−1^), *k*_*i*_ is the intraparticle diffusion rate constant (mg.g^−1^·min^−1/2^), and *C* is the thickness of the boundary layer. The values of *k*_1_ and *q*_*e*_ were calculated from the slope and intercept of the plot of ln(*q*_*e*_ − *q*_*t*_) versus *t*. While the values of *q*_*e*_ and *k*_2_ were calculated from the slope and intercept of the plot of *t/q*_*t*_ versus t, and the value of *k*_*i*_ and *C* were calculated from the plot and intercept of *q*_*t*_ versus *t*^1/2^, respectively. Table 1 summarized the values of all the three different kinetic model parameters.

**Table 1:** Kinetic model parameters of Cr(VI) adsorption on PAMAMs-GO.

C_0_	Pseudo-first-order			Pseudo-second-order			Intraparticle diffusion		
mg/L	k_1_ min ^−1^	q_e_ mg/g	R^2^	k_2_ g.mg^−1^.min^−1^	q_e_ mg/g	R^2^	k_i_ mg.g^−1^·min^−1/2^	C mg/g	R^2^
50	0.0061	8.51	0.8880	2.58 × 10^−3^	46.36	0.9999	0.1948	41.164	0.9240
100	0.0041	14.20	0.8561	1.35 × 10^−3^	74.57	0.9996	0.3903	63.973	0.9529

As shown in Figs. 1(A,B), the adsorption kinetic data of Cr(VI) on PAMAMs/GO were much more in agreement with the pseudo-second-order model than the pseudo-first-order model and intraparticle diffusion model, whose values of correlation coefficient were closer to 1 than the other two models, which were listed in Table 1. In addition, the values of *q*_*e*_ calculated from equation of the pseudo-second-order model were well agreed with the experimental values, while the *q*_*e*_ obtained from the pseudo-first-order model were poorly agreed with the experimental values. Besides, increasing the initial concentration of Cr(VI) caused a decrease in the values of *k*_2_. Furthermore, the Figs. 1(C) shows that the values of correlation coefficient *R*^2^ from the linear plot of *q*_*t*_ versus *t*^*1/2*^ in *t* = 60 to 720 min were 0.9240 and 0.9529 respectively, which represented the diffusion of Cr(VI) anions within the sorbent. Above all, the adsorption kinetics analysis clearly indicates that the mechanism of adsorption of Cr(VI) on GO /PAMAMs could be interpreted by the intraparticle diffusion model.

### Effect of initial Cr(VI) concentration, temperature and adsorption isotherm

**Figure 4:**
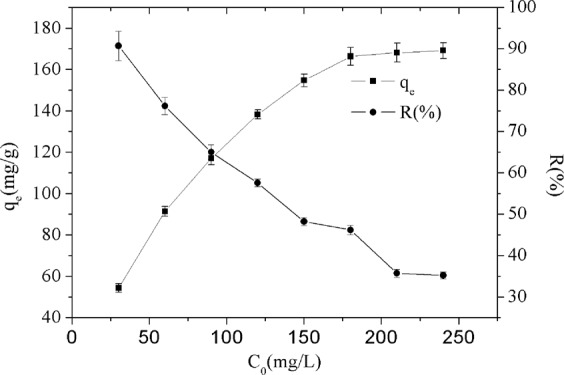
The effect of *C*_0_ on q_e_ and *R*%.

Figure 4 shows the effect of initial ion concentration on the adsorption of Cr(VI) at 303.15 K, as the initial Cr(VI) concentration increased from 30 to 240 mg/L, the equilibrium adsorption capacity keeps increasing from 54.41 to 169.09 mg/g, while the removal efficiency keeps deceasing from 90.69% to 35.22%. When the initial Cr(VI) concentration reached 180 mg/L, the adsorption capacity is almost no increasing, which means the adsorption reached saturation. This could be interpreted that when the concentration of Cr(VI) is lower than 180 mg/L, there are adequate amine groups and adsorption sites on the surface of GO/PAMAMs for removing of Cr(VI). However, when the concentration of Cr(VI) is larger than 180 mg/L, there are not enough amine groups and active sorption sites for binding with Cr(VI). Moreover, the electrostatic repulsion between negative charges of Cr(VI) oxygen anions resulted in the decline of removal percentage.

**Figure 5:**
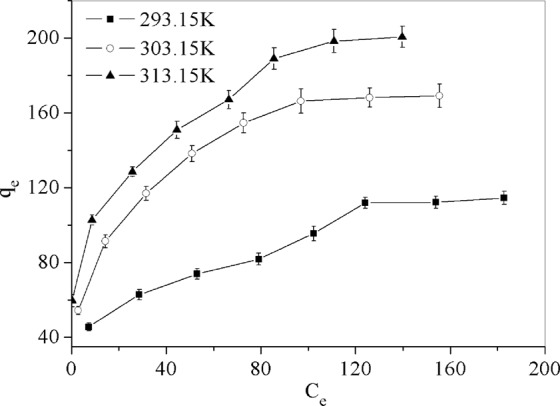
Adsorption isotherm of Cr(VI) under 3 different temperatures.

Figure 5 has shown the relation curves between the equilibrium adsorption capacity and equilibrium concentration of Cr(VI) under 3 different temperatures. The equilibrium adsorption capacities of Cr(VI) is 114.55, 169.09 and 200.56 mg/g at 293.15, 303.15 and 313.15 K, respectively, the change showed that the equilibrium adsorption capacity increase with the increasing of temperature, which means that raising the temperature from 293.15 to 313.15 K favors the adsorption of Cr(VI).

In order to obtain the saturate capacity *q*_*m*_ of Cr(VI) adsorption on PAMAMs-GO, the Langmuir and Freunlich model are widely used for fitting the adsorption isotherm data^34^. So the Langmuir and Freundlich model were applied to fit the adsorption isotherm data in our work, and the linearized forms of these models are expressed in Eqs (6) and (7)^6^, respectively.

6 $${\rm{Langmuir}}\,{\rm{model}}:\frac{{C}_{e}}{{q}_{e}}=\frac{1}{{q}_{m}}{C}_{e}+\frac{1}{{K}_{L}{q}_{m}}$$

where *K*_*L*_ (L/mg) and *q*_*m*_ (mg/g) represent the Langmuir equilibrium constant and the saturated adsorption capacity, respectively.

7 $${\rm{Freundlich}}\,{\rm{model}}:\,\mathrm{ln}\,{q}_{e}=\frac{1}{n}\,\mathrm{ln}\,{C}_{e}+\,\mathrm{ln}\,{K}_{F}$$

where *K*_*F*_ (mg/g)·(L/mg)^1/n^ is the measure of adsorption capacity and 1/*n* is the adsorption intensity.

**Figure 6:**
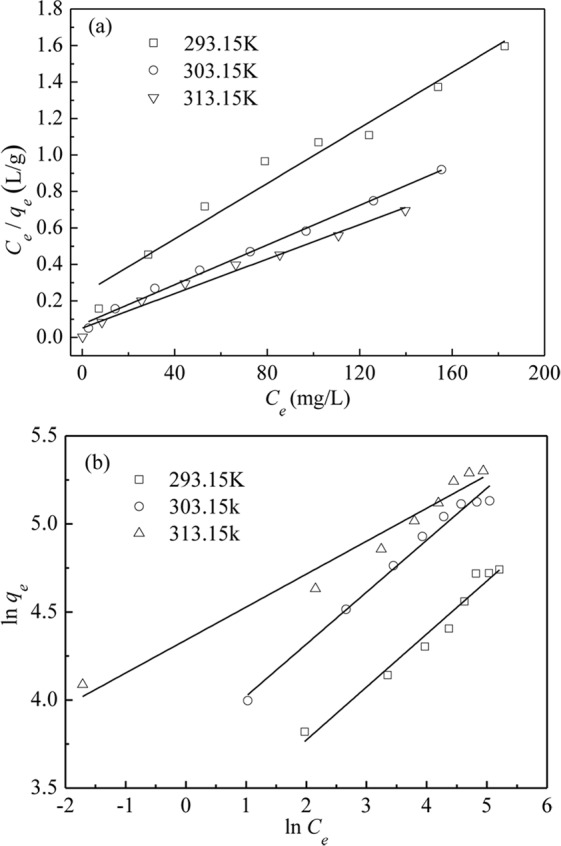
(**a**) Langmuir isotherm adsorption curve, (**b**) Freundlich isotherm adsorption curve.

The adsorption equilibrium data on PAMAMs/GO were fitted with the Langmuir and Freundlich isotherm models at 293.15, 303.15 and 313.15 K and listed in Fig. 6 and Table 2. The results showed both the correlation coefficient R^2^ of the Langmuir and Freundlich model were all very close to 1, which indicated that the two models fitted well with the adsorption isotherm data. However, the R^2^ value of Langmuir isotherm is closer to 1 than that of Freundlich isotherm, which means adsorption obeys Langmuir isotherm more than Freundlich model. Langmuir model indicates the adsorption of Cr(VI) is more likely on a homogenous surface by monolayer adsorption, and no interaction occurred between adsorption targets^28^. The essential characteristic of the Langmuir isotherm in terms of the dimensionless constant separation factor *R*_*L*_ can be calculated from Eq. (8)^35^. According to the Langmuir isotherm equation, the calculated maximum adsorption capacities for Pb^2+^ by PAMAMs/GO are 131.58, 183.82 and 211.42 mg/g at 293.15, 303.15 and 313.15 K, respectively.

8 $${R}_{L}=\frac{1}{1+{K}_{L}{C}_{0}}$$

where the value of *K*_*L*_ is the parameter listed in Table 2 and the *C*_0_ is the initial ion concentration whose value lies between 30 and 240.

**Table 2:** Parameters of Langmuir and Freundlich isotherm models.

T/K	Langmuir Constants			Freundlich Constants		
	*q*_*m*_(mg/g)	*K*_*L*_(L/g)	*R* ^2^	*K*_*F*_(L/g)	1/*n*	*R* ^2^
293.15	131.58	0.0322	0.9622	23.8106	0.301	0.9608
303.15	183.82	0.0759	0.9948	41.5031	0.295	0.9840
313.15	211.42	0.0921	0.9825	76.7521	0.187	0.9640

The values of parameter *K*_*L*_ from Langmuir model and 1/n from Frundlich model lies between 0 and 1 indicate the favorable condition for the adsorption^33^. Furthermore the values of *q*_*m*_, *K*_*L*_ and *K*_*F*_ increased with the raise of temperature reveals the endothermic nature of the adsorption process^33^. Table 3 summarizes the maximum adsorption capacities of some adsorbents for Cr(VI), which reported in recent years, it shows that the adsorption capacity of GO/PAMAMs for Cr(VI) is exceed or comparable to those of other adsorbents. So, the GO/PAMAMs can be used for treating wastewater containing Cr(VI) anions.

**Table 3:** Maximum adsorption capacities (*q*_m_) for Cr(VI) by some adsorbents.

Symbol of adsorbent	q_m_(mg/g)	pH	T/K	Isotherm	Reference
CGP	165.6	4.2	303	Freundlich	^3^
CCGP	179.2	4.2	303	Freundlich	^3^
GMA	132.5	4	298	Langmuir	^4^
Ti0.1@Ce0.9	136.98	2.0	—	Langmuir	^12^
Ti0.3@Ce0.7	142.26	2.0	—	Langmuir	^12^
GS-PPy	429.2	2	298	Langmuir	^13^
chitosan/MWCNTs-COOH	142.86	2	293	Langmuir	^20^
PTHA-4	283.29	2.5	303	Langmuir	^22^
nZVI-BC	58.82	4.0	293	Langmuir	^24^
nZVI@MG	66.2	8.0	303	Freundlich	^29^
nZVI@MG	101.0	3.0	303	Langmuir	^29^
Ppy-Fe_3_O_4_/rGO	293.3	3.0	318	Langmuir	^30^
GO/PAMAMs	211.4	2.5	313	Langmuir	This work

### Thermodynamic analysis

The influence of temperature on the thermodynamic parameters is related to the adsorption process. Several thermodynamic parameters were calculated according to Eqs (9) and (10)^31^.

9 $$\mathrm{ln}\,{K}^{0}=-\,\frac{{\rm{\Delta }}{{\rm H}}^{0}}{R}\frac{1}{T}+\frac{{\rm{\Delta }}{S}^{0}}{R}$$

10 $${\rm{\Delta }}{G}^{0}={\rm{\Delta }}{H}^{0}-T{\rm{\Delta }}{S}^{0}$$

where *K*^0^ is the adsorption distribution coefficient, which was determined from the intercept of plotting ln(*q*_*e*_/*C*_*e*_) versus *C*_*e*_ at different temperature by extroplotting *C*_*e*_ to zero according to Khan and Singh method^1^, and Δ*H*^*0*^ is the standard enthalpy change (J·mol^−1^), Δ*S*^0^ is the standard entropy change (J·mol^−1^·K^−1^), Δ*G*^0^ is the standard Gibbs free energy change, *R* is the gas constant (8.314 J·mol^−1^·K^−1^) and *T* is the absolute temperature in Kelvin (K), respectively. Δ*H*^*0*^ and Δ*S*^0^ were obtained from the slope and intercept in the curve of ln *K*^0^ versus *T*^−1^.

The graph of ln(*q*_*e*_/*C*_*e*_) versus *C*_*e*_ and ln *K*^0^ versus *T*^−1^ were plotted and showed in Figs 2 and 3, respectively. The values of ln *K*^0^, Δ*H*^*0*^, Δ*S*^0^ and Δ*G*^0^ were listed in Table 4. It is clear that there’s a highly good linear relationship between ln *K*^0^ and *T*^−1^, whose values of slope, intercept and correlation coefficient *R*^2^ determined to be −9610.81, 33.985 and 0.9846, respectively.

**Table 4:** Thermodynamic parameters for the adsorption of Cr(VI) on PAMAMs-GO.

*T*(K)	lnK^0^	Δ*H*^0^ (kJ·mol^−1^)	Δ*S*^0^ (J·mol^−1^·K^−1^)	Δ*G*^0^ (kJ·mol^−1^)
293.15	0.7850	50.859	180.25	−1.981
303.15	1.5655			−3.784
313.15	2.1156			−5.586

Generally, if the value of Δ*H*^*0*^ lies between 2.1 and 20.9 kJ·mol^−1^, it is consistent with electrostatic interaction between adsorption sites and adsorbing ions, which indicates the adsorption belongs to physical adsorption. If the value of Δ*H*^*0*^ lies between 20.9 and 418.4 kJ·mol^−1^, it reveals the adsorption involves charge sharing or transferring from adsorbent surfaces to adsorbing ions to form coordinate bonds, which indicates the adsorption owes to chemical adsorption^33^. The value of Δ*H*^*0*^ listed in Table 4 lies between 20.1 and 418.4 kJ·mol^−1^ indicates the adsorption of Cr(VI) on PAMAMs/GO belongs to chemical adsorption, meanwhile the value of Δ*H*^*0*^ = 50.859 kJ·mol^−1^ reveals the adsorption is endothermic in nature, which means that raising temperature favors the adsorption process, which agreed with the discussion about the effect of temperature change. The negative Δ*G*^0^ indicates that the adsorption of Cr(VI) onto GO/PAMAMs is a spontaneous process, and the positive Δ*S*^0^ suggests that the adsorption process is driven by entropy instead of enthalpy^36–44^.

### Effect of competitor anions

**Figure 7:**
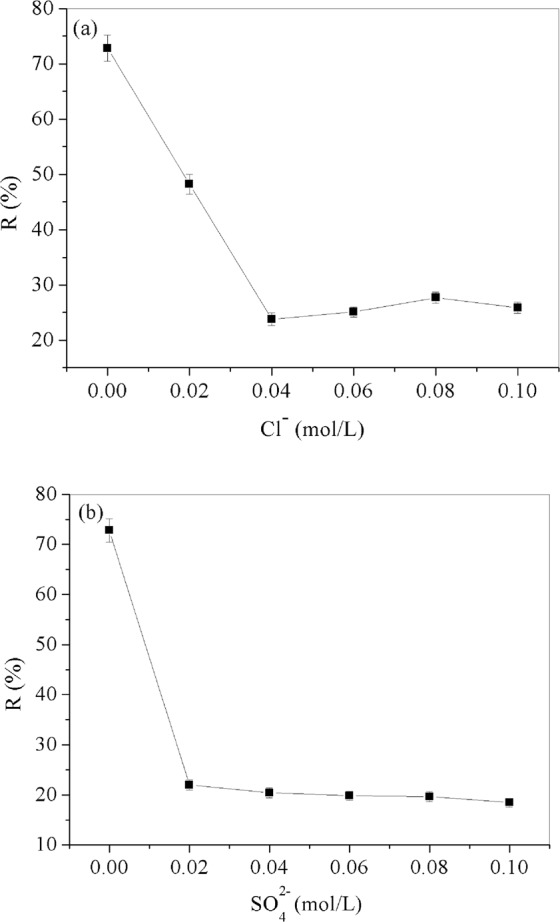
The effect of competitor anions and ion strength (**a**) Cl^−^ and (**b**) SO_4_^2−^.

As we known, the Cl^−^ and SO_4_^2−^ are widely existed in industrial wastewater, the presences of Cl^−^ and SO_4_^2−^ certainly have impact on the removal Cr(VI) on adsorbents^3,33^. Figure 7(a,b) have shown the effect of Cl^−^ and SO_4_^2−^ in the range of 0.02 ~ 0.10 mol/L on the adsorption of Cr(VI) by GO/PAMAMs. It can be seen that the existences of Cl^−^ and SO_4_^2−^ have significant influence on the adsorption of Cr(VI) on GO/PAMAMs, this may be the strong competition between anions and Cr(VI) for available adsorption sites.

### XPS analysis

**Figure 8:**
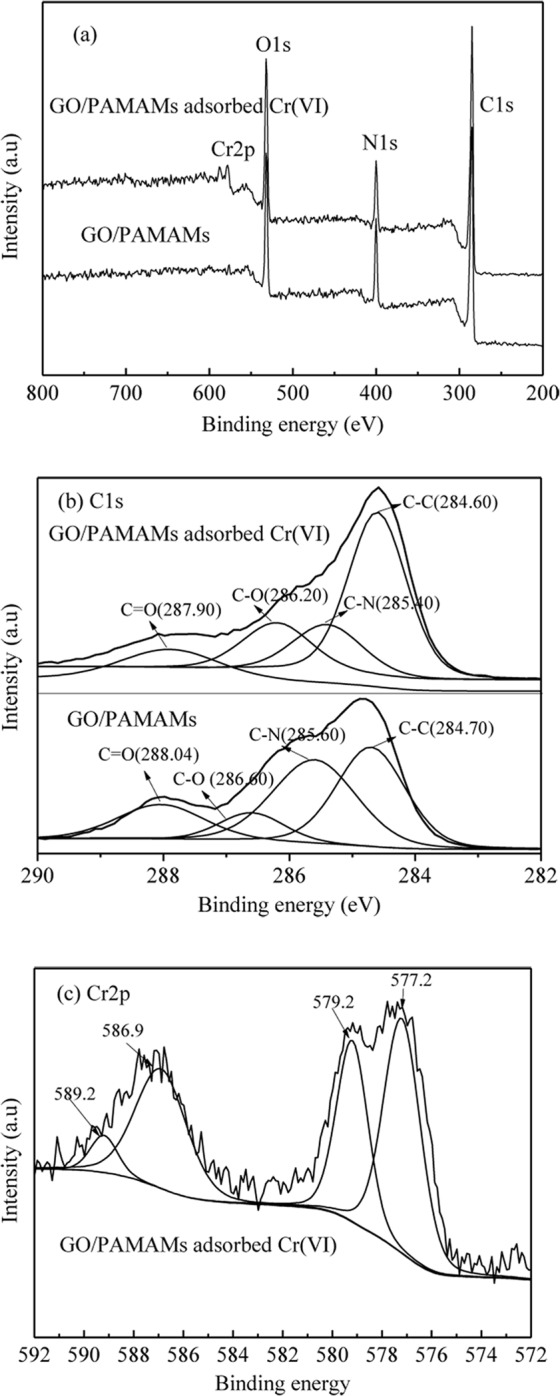
(**a**) Full scale XPS spectra and (**b**) high-resolution XPS spectra of C1s of GO/PAMAMs and GO/PAMAMs adsorbed Cr(VI), (**c**) high-resolution XPS spectra of Cr2p of GO/PAMAMs adsorbed Cr(VI).

In order to conform that Cr(VI) was actually adsorbed on GO/PAMAMs and investigate thoroughly whether the Cr(VI) was reduced to Cr(III) due to the presence of π electrons donor on GO/PAMAMs, XPS characterization was carried out for GO/PAMAMs before and after adsorbed Cr(VI). Seen from Fig. 8(a), Cr 2p peak was occurred after adsorption of Cr(VI), which conformed that Cr(VI) was actually adsorbed onto GO/PAMAMs. The composition change in GO/PAMAMs before and after adsorbed Cr(VI) by the XPS semi-quantitative surface element analysis (Table 5) further conformed the adsorption of Cr onto GO/PAMAMs. There are four different peaks at 288.04 eV, 286.60 eV, 285.60 eV and 284.70 eV (Fig. 8(b)), which corresponds to C=O, C-O, C-N and C-C of GO/PAMAMs, respectively^30^. After adsorbing Cr(VI), all the binding energies of C in GO/PAMAMs decreased a little, this may be due to the interaction between neighbor N and metal ions^45^. It has been reported that Cr(VI) can be removed from aqueous solution by adsorbents through a direct or an indirect mechanism^30,46^. In order to explore the main mechanism of Cr(VI) removal by GO/PAMAMs, the valence of Cr bound to GO/PAMAMs was also analysed by XPS characterization (Fig. 8(c)). Seen from Fig. 8(c), Cr 2p peaks can be curve-fitted with four components at binding energies of 589.2 eV, 586.9 eV, 579.2 eV and 577.2 eV, the peak components at 577.2 eV and 579.2 eV correspond to Cr 2p_3/2_ orbitals, while those at 586.9 eV and 589.2 eV correspond to Cr 2p_1/2_ orbitals. The peaks at binding energies of 579.2 eV and 589.2 eV can be regarded as the signals of Cr(VI), while the peaks at 577.2 eV and 586.9 eV can be attributed to Cr(III)^30^. The results indicate that both Cr(III) and Cr(VI) exist on GO/PAMAMs. Hence, it can be concluded that Cr(VI) was removed from aqueous solution through an indirect mechanism, and part of Cr(III) was bound to the surface of GO/PAMAMs.

**Table 5:** The atom percentage change of GO/PAMAMs before and after adsorption.

Atom (wt%)	C	N	O	Cr	Na
Before adsorption	69.95	14.31	15.74	0	0
After adsorption	67.47	12.72	17.19	1.7	0.92

It can be speculated that the removal mechanism of Cr(VI) on GO/PAMAMs consists of three steps as follows: the Cr(VI) binded to the surface of GO/PAMAMs under the electrostatic interaction between the negative charged Cr(VI) species and the protonated amine groups, then the Cr(VI) was reduced to Cr(III) with the assistance of π-electrons on the carbocyclic six-membered ring of GO in GO/PAMAMs, and then the Cr(III) was released into solution under electrostatic repulsion between the Cr(III) and the protonated amine groups, and part of Cr(III) on GO/PAMAMs was existed for the electrostatic attraction between Cr(III) and negatively charged –COO^−^ of GO/PAMAMs^30,46^.

### Regeneration study

**Figure 9:**
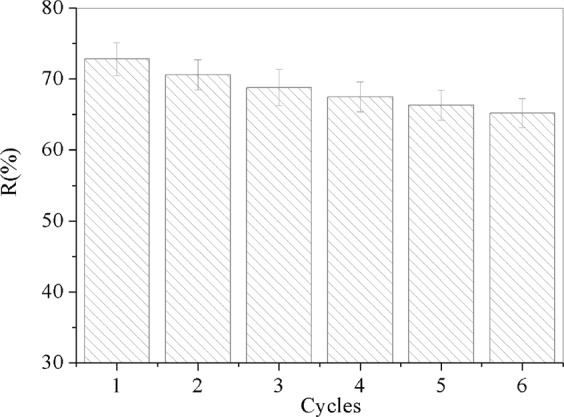
Removal efficiency of Cr(VI) of adsorption cycles.

The regeneration experiments are significant complements of adsorption studies, which could evaluate the feasibility of regeneration of the adsorbent. Figure 9 has shown the removal efficiency of Cr(VI) at each cycle after desorption with 50 mL 0.02 mol/L NaOH solution. It can be observed that the removal efficiency of Cr(VI) by GO/PAMAMs just has a little decrease after 5 times regeneration, and the removal efficiency of Cr(VI) only decreased from 72.8% to 65.2% for 100 mg/L of Cr(VI) at pH = 2.5, which indicates a good regeneration property of the adsorbent of GO/PAMAMs for removal of Cr(VI).

## Conclusions

The GO/PAMAMs is an effective adsorbent for the removal of Cr(VI) from aqueous solutions. The adsorption of Cr(VI) on GO/PAMAMs is highly pH and initial Cr(VI) concentration dependent. The optimal pH lies at 2.5, with the highest Cr(VI) removal efficiency 80.7% for C_0_ = 50 mg/L of Cr(VI) at 30 °C, while the percentage removal of Cr(VI) decreased with the increase of Cr(VI) concentration. The adsorption equilibrium experimental data fitted well with the pseudo-second-order model. The adsorption isotherm fitted well with the Langmuir model and the maximum adsorption capacity reached 211.42 mg/g at 40 °C. The thermodynamic parameters indicatethat the adsorption belongs to chemical-adsorption and the nature of spontaneous endothermic process, which is driven by entropy. The regeneration experiments showed 0.05 mol/L of NaOH solution is suitable for the regeneration of GO/PAMAMs. The XPS analysis reveals the adsorption and removal mechanism of Cr(VI) on GO/PAMAMs that first the Cr(VI) binds to the protonated amine of GO/PAMAMs, then Cr(VI) be reduced to Cr(III) with the assistance of π-electrons on the carbocyclic six-membered ring of GO in GO/PAMAMs, and then Cr(III) was released into solution under the electrostatic repulsion between the Cr(III) and the protonated amine groups.

## Experimental Section

### Materials

Chemical reagents include graphite powder, K_2_Cr_2_O_7_, K_2_S_2_O_8_, P_2_O_5_, H_2_O_2_, NH_2_CH_2_CH_2_NH_2_, methylacrylate, NaOH and HNO_3_ were purchased from Tianjin Kemiou Chemical reagent Co., Ltd, Tianjin, China. 1,5-diphenylcarbazide was purchased from Guangfu fine chemical research institute, Tianjin. All the chemical reagents are analytical grade and all the water used were double distilled water.

### Synthesis of GO/PAMAMs

The dimethylformamide (DMF) solution of Graphene Oxide (GO) was prepared via the modified Hummers method^46^. The generation 2(G2) PAMAMs were prepared according to the procedures described in the literature^47^. The GO/PAMAMs were synthesized and characterized in our reported literature^31^. Briefly, 5.0 g of G2-PAMAMs dissolved in 20 mL of absolute methanol, then mixed with 120 mL of DMF solution which contains 1.0 g of exfoliated GO with magnetic stirring in 250 mL of three-neck glass flask. After the mixture being refluxed for 24 h under 80 °C in a water bath, the warm solution was filtered and washed with 200 mL of absolute methanol until all excess G2-PAMAMs were removed from the precipitates. Finally, the colloidal sediment was transferred into a glass dish and dried in a vacuum oven at 100 °C for 12 h, thus the GO/PAMAMs composites were obtained.

### Analysis method

A stock solution containing Cr(VI) 500 mg/L was prepared by dissolving 0.7206 g of potassium dichromate with distilled water in a 500 mL of measuring flask and diluted to desired concentration, the pH of Cr(VI) solution were adjusted to desired value with 0.1 M of HNO_3_ and NaOH solution under the pH meter (Jingke, Shanghai Co. Ltd). Concentration of Cr(VI) was determined using a Ultraviolet-visible spectrophotometer (UV-2250, SHIMADZU, Japan), which analyzed through the purple red complex with 1, 5-diphenylcarbazide in acid medium at λ = 540 nm. The standard curves of Cr(VI) concentration was obtained through determining the absorbance of known concentration of Cr(VI) with 0–2.0 mg/L (seen in Fig. 4), the linear equation of absorbance (A) versus Cr(VI) concentration (C, mg/L) was calculated as follow: A = 0.31693 C + 0.00296, the scope of A is 0–0.7 and the correlation coefficient of the standard curve is 0.9973.

### Batch adsorption experiments

The batch experiments of investigating influence factors for adsorption were performed in a series of 100 mL of Cr(VI) solution in 250 mL of stand-up bottom flask under the conditions of constant magnetic stirring speed, adsorbent dosage 0.05 g and contact time 24 h. The effect of different initial aqueous pH values were investigated in the range of 1.5–7.0 with initial Cr(VI) concentration 50 mg/L at 303.15 K, the optimum pH value medium was adopted for all the following experiments. The effect of initial Cr(VI) concentration was performed in the range of 30–240 mg/L. the adsorption isotherm experiments were performed by varying initial Cr(VI) concentration from 30–240 mg/L at three different temperatures of 293.15, 303.15, and 313.15 K within 6 h, respectively. The batch adsorption equilibrium experiments were performed in a 1000 mL three-neck bottom flask where 500 mL Cr(VI) solution mixed with 0.5 g adsorbent by varying the initial Cr(VI) concentration (50, 100 mg/L) at 303.15 K and constant stirring speed in the water bath, 2 mL of supernatant sample was taken out at various time intervals (10–720 min) and filtered immediately through 0.45 μm filter paper. The residual concentration of Cr(VI) was determined, then the equilibrium adsorption capacity (*q*_*e*_, mg/g) and removal efficiency (*R*) of Cr(VI) were calculated according to Eqs (11) and (12)^4^, respectively:

11 $${q}_{e}=\frac{({C}_{0}-{C}_{e})V}{m}$$

12 $$R \% =\frac{{C}_{0}-{C}_{e}}{{C}_{0}}\times 100 \% $$

where *C*_0_ and *C*_*e*_ represent the initial and equilibrium concentration (mg/L), respectively, *V* represents the volume of solution (L), *m* represents the mass of PAMAMs/GO (g).

### Regeneration studies

Regeneration studies of PAMAMs/GO was performed four times for initial *C*_0_ = 50 mg/L of Cr(VI) at 303.15 K after desorption by 0.05 M of NaOH solution. After Cr(VI) desorption, the PAMAMs/GO was air dried and dissolved in 100 mL Cr(VI) solution for 6 h adsorption, then the residual concentration of Cr(VI) was determined.

All the experiments were duplicated and only the average values were chosen for plotting. Furthermore, the maximum errors were being controlled within 5%.

## Supplementary information

Supplementary Material
