# Supplementary material for: Adsorption mechanism of Cr(VI) onto GO/PAMAMs composites
Source: Sci Rep. 2019 Mar 6;9:3663. doi: 10.1038/s41598-019-40344-9 (PMC6403246; doi:10.1038/s41598-019-40344-9)
Supplement: Supplementary file 1 — Supplementary Material [file 41598_2019_40344_MOESM1_ESM.docx]

Adsorption mechanism of Cr(VI) onto GO/PAMAMs composites

*Han Liu, Fan Zhang***^[[1]](#footnote-2)^*** *,* *Zhiyuan Peng*

**Supplementary Material**

Figs.1 (a) Pseudo-first-order kinetic model for adsorption of Cr(VI), (b) Pseudo-second-order kinetic model for adsorption of Cr(VI), (c) Intraparticle diffusion model for adsorption of Cr(VI).

Figs.2 Relationship curve between lnK_d_ and C_e_

Figs.3 Relationship curve between lnK_0_ and T^-1^

Figs. 4 Calibration curve of Cr(VI) in acid solution with K_2_Cr_2_O_7_ at542 nm

1. * 2011 Cooperative Innovation Center of industrial technology for manganese，zinc and vanadium of Hunan Province; Hunan Provincial Engineering Laboratory of Integrated Control and Remediation of Heavy Metal Pollution from Mn-Zn Mining; National Demonstration Center for Experimental Chemistry Education, Jishou University, Jishou, Hunan, 416000, China. Correspondence and requests for materials should be addressed to Zhang F. ([chemfzhang@163.com](mailto:chemfzhang@163.com)). [↑](#footnote-ref-2)
